# Evaluating Participation Modes in Peracetylated Glycosyl Cations

**DOI:** 10.1021/acs.orglett.5c04756

**Published:** 2026-01-02

**Authors:** Niklas Geue, Kim Greis, Sabrina Omoregbee-Leichnitz, Carla Kirschbaum, Gerard Meijer, Gert von Helden, Peter H. Seeberger, Mateusz Marianski, Kevin Pagel

**Affiliations:** † Institute of Chemistry and Biochemistry, 9166 Freie Universität Berlin, Altensteinstraße 23a, 14195 Berlin, Germany; ‡ Department of Molecular Physics, 28259 Fritz-Haber-Institut der Max-Planck-Gesellschaft, Faradayweg 4−6, 14195 Berlin, Germany; § Max Planck Institute of Colloids and Interfaces, Am Mühlenberg 1, 14476 Potsdam, Germany; ∥ Department of Chemistry, 5924 Hunter College, The City University of New York, New York, New York 10065, United States

## Abstract

Neighboring group participation and remote participation are fundamental concepts in carbohydrate chemistry and are commonly applied to achieve stereocontrol in glycosylation reactions. The corresponding intermediates can be glycosyl cations, which are challenging to characterize due to their short-lived nature. Using gas-phase infrared spectroscopy supported by density functional theory calculations, we directly assess and rank the participation modes of acetyl protecting groups in peracetylated glucosyl, galactosyl, and mannosyl cations, showing that neighboring group participation and hence the formation of C2-dioxolenium ions is found experimentally in all three cases. Energetic ranking of theoretical structures *in vacuo* revealed differences in the remote participation preferences depending on the hexose, showing that C3-dioxolenium ions are preferred for glucose and particularly mannose, whereas C4-dioxolenium ions are most stable in the case of galactose. These findings contribute to our fundamental understanding of protecting group participation and could facilitate glycosylation strategies in the future.

## Graphical abstract

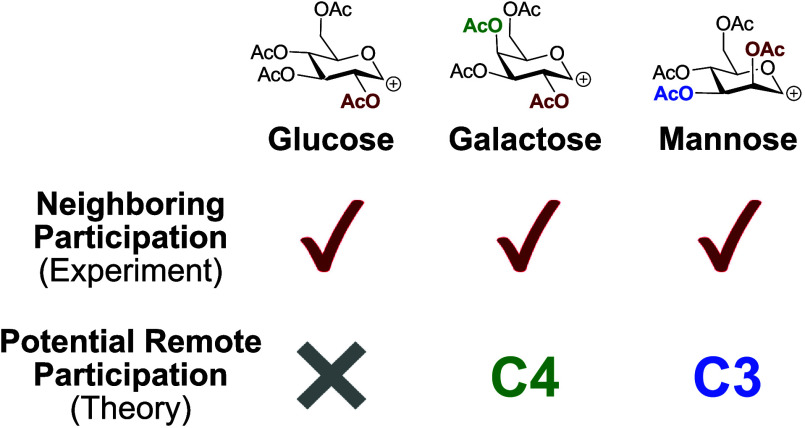

The formation of glycosidic bonds is at the heart of carbohydrate chemistry, and achieving regio- and stereoselectivity in such glycosylation reactions is challenging.^1^ While regiochemistry can be controlled through various protecting group strategies, the stereochemistry at the anomeric center (formation of α- vs β-anomer) is more difficult to influence. For the formation of 1,2-*trans* glycosidic bonds (e.g., β-galactosylation), the installation of ester protecting groups at C2 is known to yield high selectivity. This so-called neighboring group participation (Figure 1), also referred to as anchimeric assistance, can follow the formation of cyclic 1,2-*cis*-dioxolenium ions, which shield the *cis*-side and shift the selectivity toward a *trans*-attack of the nucleophile.^2–5^ Common strategies for the opposite outcome, the formation of 1,2-*cis* glycosidic bonds (e.g., α-galactosylation), include the use of specific activators^6^ or chiral auxiliaries^7^ or the installation of participating protecting groups at other hydroxy groups, for example, at C3, C4, and in principle at C6.^8^ This effect is known as remote participation (Figure 1) and can lead to the formation of C3- or C4- (or C6-) dioxolenium ions, and depending on the sugar moiety potentially results in the shielding of the *trans*-side and therefore a shift to *cis*-stereoselectivity.^9–11^

**Figure 1:**
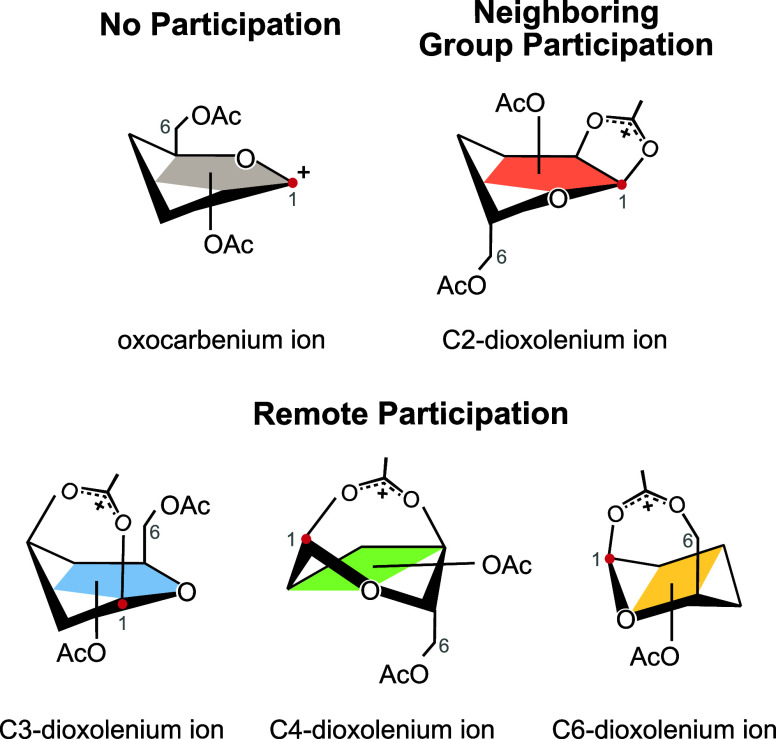
Potential glycosyl cation structures for peracetylated galactose, showcasing no participation (oxocarbenium, gray), neighboring group participation (C2-dioxolenium ion, red), and remote participation (C3- (blue), C4- (green) and C6-dioxolenium ions (yellow)). The anomeric center is labeled as C1 and depicted with a red point.

Both types of participation often occur through glycosyl cations that are short-lived and challenging to characterize.^12,13^ Various NMR-based strategies have been introduced to stabilize and characterize these intermediates, including their stabilization with super acids prior to NMR,^14^ as well as the use of low-temperature NMR^15^ and chemical exchange saturation NMR.^16,17^ A suitable alternative is to generate glycosyl cations through fragmentation in a mass spectrometer,^18^ and to characterize their structure *in situ* with gas-phase infrared (IR) spectroscopy.^19–21^ This method involves the vibrational excitation of ions with IR light at user-defined, varying wavenumbers, resulting in fragmentation or another system response when the radiation is in resonance with the vibrational modes of the ions. This allows the measurement of experimental IR spectra of gas-phase ions that can be compared to those generated from density functional theory (DFT) optimized structures.^22^

Previous work using gas-phase IR spectroscopy showed that neighboring group^3–5,23^ and remote participation^9,10,24–26^ occur for acyl groups in glycosyl cations, and a fundamental question that remains is whether and to what extent the different dioxolenium ions (C2-, C3-, C4-, and C6-) are preferred for different sugar building blocks. It is generally known that remote participation is less efficient than neighboring group participation,^27^ and for example, neighboring participation of benzoyl groups is favored over remote participation of Fmoc or benzyl groups in gaseous fluorinated glucosyl cations.^5^ Hansen et al. characterized the glycosyl cations of mannose, galactose and glucose building blocks with varying positions (C3, C4, and C6) of acetyl (Ac) protecting groups, benchmarking the preference for remote participation depending on Ac group position and sugar moiety.^25^ More recently, Boltje and co-workers studied C3,C4-diacetylated hexose building blocks using isotope labeling and isomer population analysis.^28^ Their results showed that C3-dioxolenium ions are preferred for mannose, whereas galactose (61%:35%) and glucose (40%:60%) show both C3-dioxolenium and C4-rearranged ions with different ratios, respectively. To date, a systematic study that energetically ranks the preference of C2-, C3-, C4- and C6-participation of the same protecting group within the same ion is lacking.

Here, we investigate the participation modes and dioxolenium ions that occur with acetyl protecting groups, by characterizing the glycosyl cations of peracetylated glucose, galactose, and mannose (**2,3,4,6-Ac-Glc**, **2,3,4,6-Ac-Gal**, and **2,3,4,6-Ac-Man**). We observed the formation of C2-dioxolenium ions in all cases experimentally, which was further supported by the energetic trends of the DFT optimized structures. Among the remote participation modes, the ranking *in vacuo* revealed a preference for C3-dioxolenium ions in glucose and particularly mannose, whereas C4-dioxolenium ions are theoretically favored for galactose.

The glycosyl cations of all three peracetylated building blocks were structurally characterized by cryogenic gas-phase IR spectroscopy and DFT calculations. The respective thioglycoside precursors were transferred to the gas phase via nanoelectrospray ionization and subjected to in-source fragmentation, yielding glycosyl cations (Figure S1). The experimental IR spectra of glycosyl cations consist of two major regions: the fingerprint region (1000–1400 cm^–1^) and the functional group region (1400–1800 cm^–1^). While the former mainly involves C–O and C–C stretching as well as C–H bending vibrations, the region of functional groups is largely populated by carbonyl stretching ν­(C=O) as well as symmetric and antisymmetric dioxolenium ν­(O–C–O^+^) vibrations.^29^ Glycosyl cations were characterized by comparing the experimental IR spectra to the harmonic frequencies of candidate structures, which were obtained through rigorous conformational search. The sampling included dioxolenium structures that show neighboring (C2-dioxolenium) and remote participation (C3-, C4-, and C6-dioxolenium), as well as oxocarbenium ions without any participation (Tables S1–S3, Figures S2–S4).

For **2,3,4,6-Ac-Glc**, C2-dioxolenium ions are the lowest in energy (Table S1) and have the best agreement with the experimental spectrum (Figure 2). This includes the C=O stretching in the region between 1730 and 1770 cm^–1^, as well as the symmetric (1510 cm^–1^) and antisymmetric (1555 cm^–1^) C2-dioxolenium stretching vibrations. Further, the fingerprint region between 1000 and 1400 cm^–1^ resembles the simulated spectrum of the C2-dioxolenium structure clearly best (Figure 2). Although the region between 1700 cm^–1^ and 1750 cm^–1^ is not fully resolved and shows multiple bands, both the high energies and the absence of characteristic dioxolenium bands between 1450 and 1500 cm^–1^ suggest that no remote participation occurs in **2,3,4,6-Ac-Glc** through C3, C4 or C6. The relative stability ranking based on DFT energies follows the order C2-dioxolenium > C3-dioxolenium > C4-dioxolenium > C6-dioxolenium > oxocarbenium.

**Figure 2:**
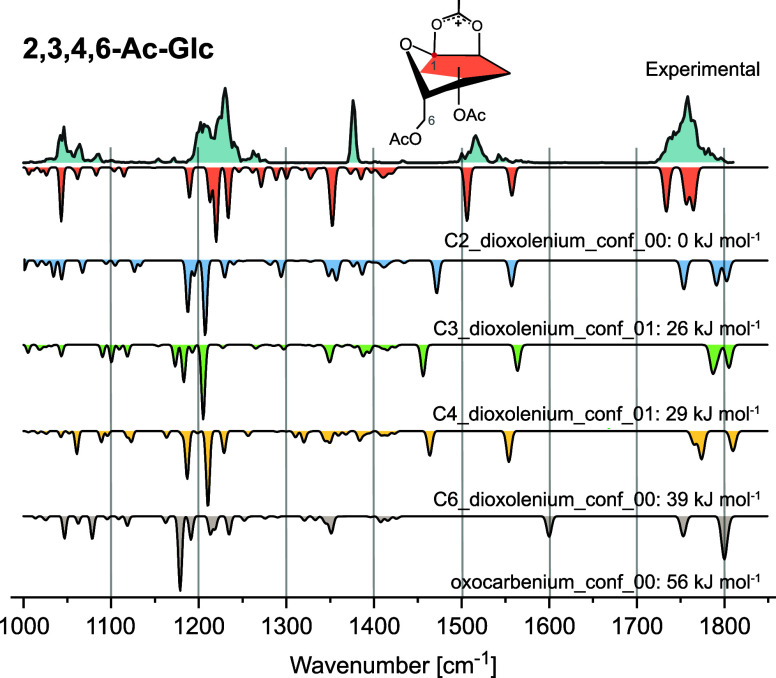
Cryogenic infrared spectrum of the **2,3,4,6-Ac-Glc** glycosyl cation. Computed IR spectra are shown as inverted traces for structures with neighboring participation (C2-dioxolenium: red), remote acyl participation (C3-dioxolenium: blue; C4-dioxolenium: green; and C6-dioxolenium: yellow) and no participation (oxocarbenium: gray). The best agreement is found with C2-dioxolenium ions, and their structure is shown. The absence of dioxolenium bands between 1450 and 1500 cm^–1^ excludes the occurrence of remote participation modes.

The glycosyl cation **2,3,4,6-Ac-Gal** similarly matches best with C2-dioxolenium cations through assignments of the bands discussed above, and here a mixture of C2-dioxolenium conformers is likely present simultaneously (Figure 3). While DFT-based energetics also favor neighboring participation for the galactosyl cation (Table S2), the C3- and particularly C4-dioxolenium ions are closer in energy than for the glucosyl case. Due to the occurrence of more bands in the diagnostic region, e.g., at ca. 1480 cm^–1^, and the small difference in energetics, the presence of C4- and/or C3-dioxolenium ions cannot be fully excluded. The relative rankings of DFT energies for remote participation are different from the glucosyl cation, with the C4-dioxolenium ions (11 kJ mol^–1^) preferred over C3-dioxolenium (17 kJ mol^–1^).

**Figure 3:**
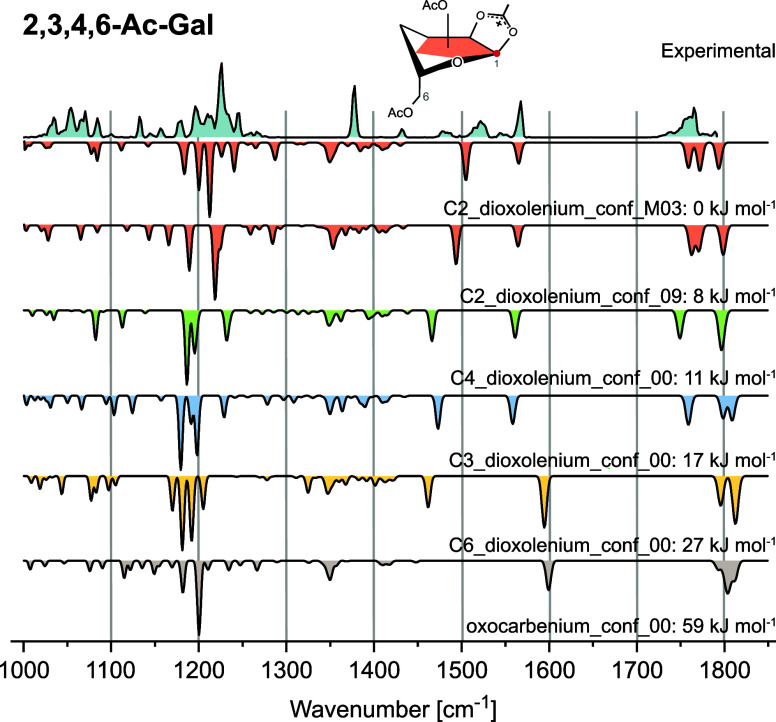
Cryogenic infrared spectrum of the **2,3,4,6-Ac-Gal** glycosyl cation. Computed IR spectra are shown as inverted traces for structures with neighboring participation (C2-dioxolenium: red), remote acyl participation (C3-dioxolenium: blue; C4-dioxolenium: green; and C6-dioxolenium: yellow) and no participation (oxocarbenium: gray). The best agreement is found with a mixture of C2-dioxolenium conformers (lowest energy conformer shown), with some traces of C3- and C4-dioxolenium ions potentially present. The latter two cannot be excluded due to additional experimental bands, e.g., at 1480 cm^–1^.

In the case of **2,3,4,6-Ac-Man**, energetics again favor C2-dioxolenium ions (Table S3), and a combination of multiple energetically low-lying conformers resembles the experimental spectrum well (Figure 4). The dioxolenium bands between 1500 cm^−1^ and 1600 cm^–1^ are diagnostic for neighboring participation and exclude the presence of other structures, for which the split between the two dioxolenium bands is larger than observed. Specifically, C3 remote participation is unlikely due to the absence of the symmetric dioxolenium stretching mode in the experimental spectrum (1475 cm^–1^). The relative energetics suggest that C3-dioxolenium ions are far more likely than C4- and C6-dioxolenium ions, which lie ca. 30 kJ mol^–1^ higher in energy.

**Figure 4:**
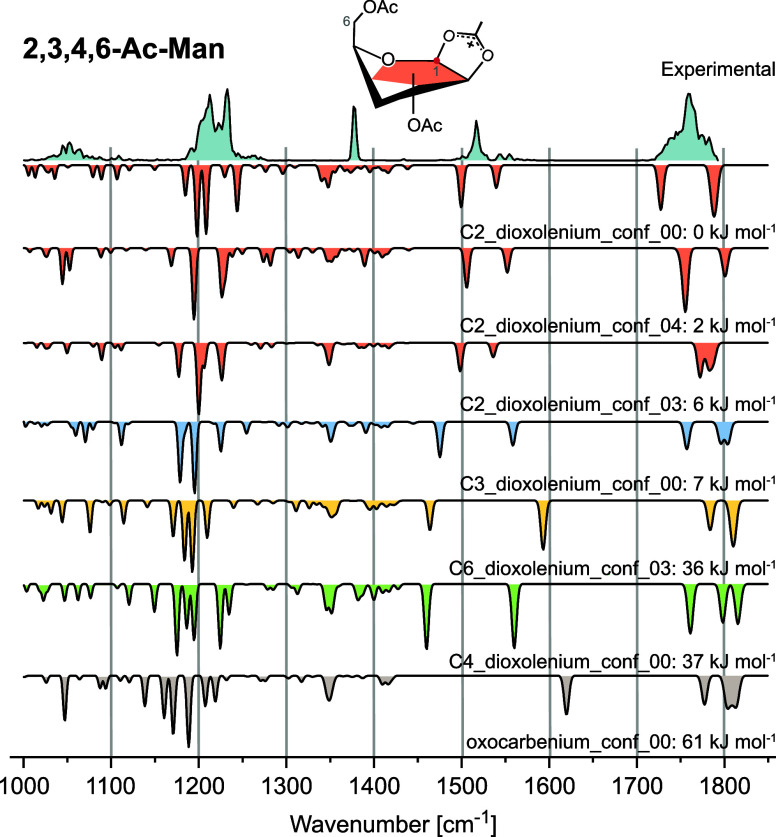
Cryogenic infrared spectrum of the **2,3,4,6-Ac-Man** glycosyl cation. Computed IR spectra are shown as inverted traces for structures with neighboring participation (C2-dioxolenium: red), remote acyl participation (C3-dioxolenium: blue; C4-dioxolenium: green; and C6-dioxolenium: yellow) and no participation (oxocarbenium: gray). The best agreement is found with a mixture of C2-dioxolenium conformers, the lowest energy structure of which is shown. The absence of dioxolenium bands between 1450 and 1500 cm^–1^ excludes the occurrence of remote participation modes.

Our experimental results show that neighboring group participation, evidenced by the formation of a five-membered, cyclic C2-dioxolenium ion, is energetically preferred for all three studied hexoses (glucose, galactose, and mannose) over remote participation of any kind (C3-, C4-, and C6-dioxolenium ions) and no participation. This agrees with a previously published IRMPD spectrum of **2,3,4,6-Ac-Man**, where C2-dioxolenium ions were found experimentally and modelling also suggested this preference.^3^ The dominance of neighboring group participation over remote participation is not surprising and agrees with general experiences in synthetic glycochemistry.^5^

Our DFT-based ranking on C2-, C3-, C4-, and C6-dioxolenium as well as oxocarbenium ions gives further insights into the preference of participation of acyl groups (Figure 5). Other studies have previously ranked and elucidated relative preferences across participation sites,^25,28^ and here we study the same protecting group at all four hydroxy groups, enabling the elucidation of the intrinsic preference for acyl participation at different sites. The energetics show a preference for C2-dioxolenium ions in each case, as found experimentally (Figures 2–4); however, they also show that all types of remote participation (C3, C4, and C6) are favored over no participation (oxocarbenium ions, Figure 5). It can hence be assumed that this ranking, neighboring group participation over remote participation over no participation, is general for glycosyl cations of monosaccharides. However, the energy differences between neighboring group participation and remote participation through C3 (mannose and to a lesser extent glucose) and C4 (galactose) are surprisingly small. This barrier could potentially be overcome, e.g., through the introduction of electron-donating protecting groups at C3/C4 or electron-withdrawing groups at C2, which would enable remote participation even when C2-participating groups are present.

Ranking the preferences of remote participation sites is more subtle and depends on the hexose studied. C6-Acyl protected sugars have previously shown no remote participation, and the relatively high energetics of C6-dioxolenium ions found here support these findings.^10,25^ The main reason for the absence of remote participation for C6- (and some C4-acyl protected sugars), however, is the occurrence of ring-opening rearrangements, which are assumed to be gas-phase artifacts without relevance for solution chemistry.^9,25,30^ The fact that no C6-dioxolenium ions are usually found is therefore not due to the intrinsically unfavored energetics; in fact, it is favored over no participation; but largely attributable to the preference for energetically low-lying gas-phase rearrangements.

Practically, remote participation occurs only through C3 and C4, and the relative preference depends on the hexose. ter Braak et al. recently used isotope labeling and isomer population analysis to experimentally rank the remote participation in 3,4-diacetylated glycosyl cations.^28^ In our study, we found a slight energetic preference for C3- over C4-dioxolenium ions for glucose (Figure 5), whereas ter Braak et al. additionally considered rearrangement reactions and found 40% C3-dioxolenium and 60% C4-rearranged structures. The case of galactose shows a preference for C4- over C3-dioxolenium ions in our theoretical data (17 kJ mol^–1^ vs 11 kJ mol^–1^, Table S2 and Figure 5); however, ter Braak et al. found C3- and C4-dioxolenium ions in ratios of 61%:35%, in agreement with their calculations.^28^ This contrast could be related to differences in conformational sampling,^31^ experimental temperatures and hence populations, or to (de)­stabilizing interactions of the C6-acyl group with C3- or C4-dioxolenium ions in our data. The latter was in fact found to be the case for C4-dioxolenium ions, which could explain their higher stability compared to the 3,4-diacetylated cation studied by ter Braak et al., where no C6-acyl group is present. For the mannosyl cation, we find a strong preference for C3-remote participation (7 kJ mol^–1^ vs 37 kJ mol^–1^ for C4-dioxolenium ions, Table S3), in agreement with ter Braak et al., who exclusively found C3-dioxolenium ions experimentally,^28^ and de Kleijne et al., who observed C3-dioxolenium mannosyl ions using exchange saturation transfer NMR spectroscopy.^32^

While the remote participation ranking is individual to each of the three hexoses, a global correlation with the ring-size of the dioxolenium intermediate was found (Figure 5), as previously hypothesized to be a stability-determining factor for Ferrier cations.^30^ C2-dioxolenium ions are five-membered rings and the most stable ion for all three building blocks, largely followed by C3-dioxolenium ions (six-membered rings) and C4-dioxolenium ions (seven-membered rings, Figure 1). C6-dioxolenium ions are also seven-membered rings and are the least stable species based on participation. Whether the ring size has a direct impact on stability preferences warrants further investigations. Other factors that likely play a role are the ring strain and conformational landscape of the respective dioxolenium rings, as previously studied for the three different hexoses.^25^ This could, for example, explain the preferred stability of the C4-dioxolenium ion for galactose, despite forming a larger ring than the respective C3-dioxolenium ion (Figure 5).

**Figure 5:**
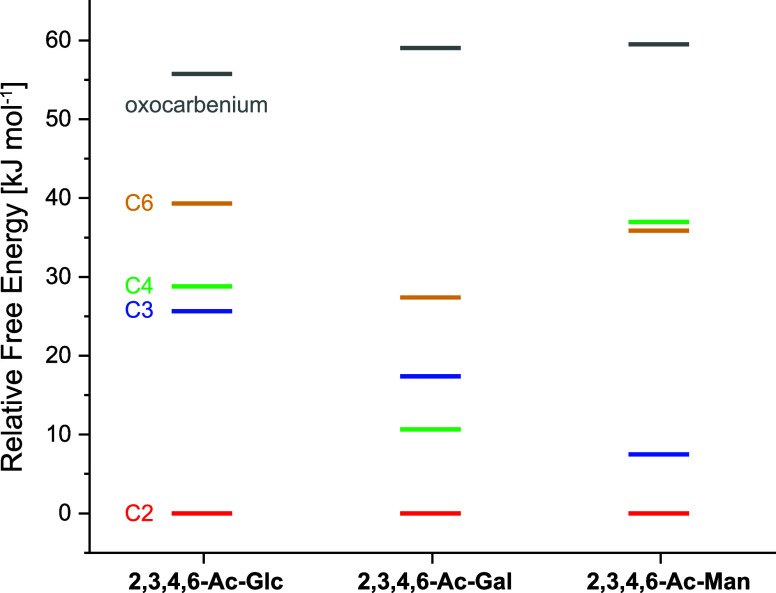
Relative free energies of the most stable conformer of C2- (red), C3- (blue), C4- (green), and C6-dioxolenium ions (yellow) as well as oxocarbenium ions (gray) for **2,3,4,6-Ac-Glc**, **2,3,4,6-Ac-Gal**, and **2,3,4,6-Ac-Man**. C2-dioxolenium ions are always most stable and set at 0 kJ mol^–1^, whereas oxocarbenium ions are highest in energy for all three hexoses. For ions indicating remote participation (C3-, C4- and C6-dioxolenium ions), different energetic trends were found for glucose (C3 < C4 < C6), galactose (C4 < C3 < C6), and mannose (C3 < C6 ≈ C4). For glucose, only C2-dioxolenium ions are feasible, whereas C4-dioxolenium ions are possible for galactose due to the axial orientation at C4. In the case of mannose, C3-dioxolenium ions can potentially occur due to the unusual axial arrangement at C2.

We further aimed to relate our findings to glycosylation reactions in solution and assessed the preference for the different participation modes in a range of solvents using implicit solvation models (Figures S5–S7).^33^ The results show that C2-dioxolenium ions are most stable for all three monosaccharides and in all solvents assessed; however, the energy difference to remote participation modes varies with the solvent and its dielectric constant. Particularly the energetic difference between C2- and C3-dioxolenium ions (for **2,3,4,6-Ac-Man,** Figure S7), and between C2- and C4-dioxolenium ions (for **2,3,4,6-Ac-Gal**, Figure S6) increase for solvents with higher permittivity, suggesting that the small energy gaps *in vacuo* do not necessarily hold in solution.

Taken together, neighboring participation through the C2 acyl group is experimentally observed and energetically lowest for the three peracetylated glycosyl cations studied.^34^ Based on the energies of the DFT optimized structures *in vacuo* for each hexose, we ranked the preference for the different types of participation following the order C2 ≫ C3 > C4 > C6 > oxocarbenium (glucose), C2 > C4 > C3 > C6 ≫ oxocarbenium (galactose), and C2 > C3 ≫ C6 ≈ C4 ≫ oxocarbenium (mannose). This systematic characterization of different acyl-participation sites in the same ion is fundamentally interesting for the formation of 1,2-*cis* glycosidic bonds in glycosylation reactions. Specifically, the small energetic differences between C2- and C3-dioxolenium ions (for mannose) as well as C2- and C4-dioxolenium ions (for galactose) suggest that remote participation might be possible even in the presence of C2-participating protection groups, for example, through the introduction of electron-withdrawing or electron-donating substituents.

## Supplementary Material



## Data Availability

Raw data for mass spectrometry, gas-phase infrared spectroscopy measurements, and density functional theory calculations was deposited on Figshare (https://figshare.com/articles/dataset/Supplementary_Dataset_for_Evaluating_C2-Neighbouring_and_C3-_C4-_and_C6-Remote_Participation_in_Peracetylated_Glycosyl_Cations_/29975410?file=60000899).

## Notes

Experimental details, mass spectra, DFT structures and energetics (PDF)

## ⊥

Department of Chemistry and Applied Biosciences, ETH Zürich, 8093 Zürich, Switzerland

## ◆

Lonza, 3930 Visp, Switzerland.

## ∇

Kavli Institute for Nanoscience Discovery, University of Oxford, Oxford OX1 3QU, UK.

## References

[1] Chatterjee S., Moon S., Hentschel F., Gilmore K., Seeberger P. H. (2018). An Empirical
Understanding of the Glycosylation Reaction. J. Am. Chem. Soc..

[2] Capon, B. ; McManus, S. P. Neighboring Group Participation; Springer US: Boston, MA, 1976. 10.1007/978-1-4684-0826-3

[3] Elferink H., Severijnen M. E., Martens J., Mensink R. A., Berden G., Oomens J., Rutjes F. P. J. T., Rijs A. M., Boltje T. J. (2018). Direct
Experimental Characterization of Glycosyl Cations by Infrared Ion
Spectroscopy. J. Am. Chem. Soc..

[4] Mucha E., Marianski M., Xu F.-F., Thomas D. A., Meijer G., von Helden G., Seeberger P. H., Pagel K. (2018). Unravelling the Structure
of Glycosyl Cations via Cold-Ion Infrared Spectroscopy. Nat. Commun..

[5] Greis K., Kirschbaum C., Fittolani G., Mucha E., Chang R., von Helden G., Meijer G., Delbianco M., Seeberger P. H., Pagel K. (2022). Neighboring Group Participation of
Benzoyl Protecting Groups in C3- and C6-Fluorinated Glucose. Eur. J. Org. Chem..

[6] Nigudkar S. S., Demchenko A. V. (2015). Stereocontrolled
1,2-Cis Glycosylation as the Driving
Force of Progress in Synthetic Carbohydrate Chemistry. Chem. Sci..

[7] Kim J.-H., Yang H., Boons G.-J. (2005). Stereoselective
Glycosylation Reactions
with Chiral Auxiliaries. Angew. Chem., Int.
Ed..

[8] Hahm H. S., Hurevich M., Seeberger P. H. (2016). Automated Assembly of Oligosaccharides
Containing Multiple Cis-Glycosidic Linkages. Nat. Commun..

[9] Greis K., Leichnitz S., Kirschbaum C., Chang C.-W., Lin M.-H., Meijer G., von Helden G., Seeberger P. H., Pagel K. (2022). The Influence of the
Electron Density in Acyl Protecting Groups on
the Selectivity of Galactose Formation. J. Am.
Chem. Soc..

[10] Marianski M., Mucha E., Greis K., Moon S., Pardo A., Kirschbaum C., Thomas D. A., Meijer G., von Helden G., Gilmore K., Seeberger P. H., Pagel K. (2020). Remote Participation
during Glycosylation Reactions of Galactose Building Blocks: Direct
Evidence from Cryogenic Vibrational Spectroscopy. Angew. Chem., Int. Ed..

[11] Dahlmann F., Griesbach C. E., Torres-Boy A. Y., von Helden G., Peczuh M. W., Pagel K., Greis K. (2025). Direct Experimental
Characterization of a Sialyl Cation. Chem. Eur.
J..

[12] Braak F. t., Elferink H., Houthuijs K. J., Oomens J., Martens J., Boltje T. J. (2022). Characterization of Elusive Reaction Intermediates
Using Infrared Ion Spectroscopy: Application to the Experimental Characterization
of Glycosyl Cations. Acc. Chem. Res..

[13] Franconetti A., Ardá A., Asensio J. L., Blériot Y., Thibaudeau S., Jiménez-Barbero J. (2021). Glycosyl Oxocarbenium
Ions: Structure, Conformation, Reactivity, and Interactions. Acc. Chem. Res..

[14] Lebedel L., Ardá A., Martin A., Désiré J., Mingot A., Aufiero M., Aiguabella Font N., Gilmour R., Jiménez-Barbero J., Blériot Y., Thibaudeau S. (2019). Structural and Computational Analysis
of 2-Halogeno-Glycosyl
Cations in the Presence of a Superacid: An Expansive Platform. Angew. Chem., Int. Ed..

[15] Upadhyaya K., Subedi Y. P., Crich D. (2021). Direct Experimental
Characterization
of a Bridged Bicyclic Glycosyl Dioxacarbenium Ion by 1H and 13C NMR
Spectroscopy: Importance of Conformation on Participation by Distal
Esters. Angew. Chem., Int. Ed..

[16] de
Kleijne F. F. J., Elferink H., Moons S. J., White P. B., Boltje T. J. (2022). Characterization of Mannosyl Dioxanium Ions in Solution
Using Chemical Exchange Saturation Transfer NMR Spectroscopy. Angew. Chem., Int. Ed..

[17] de
Kleijne F. F. J., ter Braak F., Piperoudis D., Moons P. H., Moons S. J., Elferink H., White P. B., Boltje T. J. (2023). Detection and Characterization of Rapidly Equilibrating
Glycosylation Reaction Intermediates Using Exchange NMR. J. Am. Chem. Soc..

[18] Geue N., Safferthal M., Pagel K. (2025). Collision-Induced Fragmentation of
Oligosaccharides: Mechanistic Insights for Mass Spectrometry-Based
Glycomics. Angew. Chem., Int. Ed..

[19] Chang C.-W., Wehner D., Prabhu G. R. D., Moon E., Safferthal M., Bechtella L., Österlund N., Vos G. M., Pagel K. (2025). Elucidating
Reactive Sugar-Intermediates by Mass Spectrometry. Commun. Chem..

[20] Geue, N. ; Walton-Doyle, C. ; Renzi, E. ; Bejoy, M. ; Pagel, K. Advanced Mass Spectrometry Techniques for the Characterization of Carbohydrates. In Complex Carbohydrates in Health and Disease; Handbook of Experimental Pharmacology; Springer Nature: Berlin, Heidelberg, 2025; pp 73–108. 10.1007/164_2025_749 40323419

[21] Prabhu G. P. D., Götze M., Greis K., Torres-Boy A., Safferthal M., Strzelecka D., Kirschbaum C., Deshpande N. D., Geue N., Meijer G., von Helden G., Pagel K. (2025). Cryogenic Gas-Phase Infrared Ion Spectroscopy of Ultraviolet-Induced
Nucleotide Photoproducts. Anal. Chem..

[22] Geue N., Prabhu G. R. D., Renzi E., Walton-Doyle C., Meijer G., von Helden G., Pagel K. (2025). Distinguishing Isomeric
Caffeine Metabolites through Protomers and Tautomers Using Cryogenic
Gas-Phase Infrared Spectroscopy. Anal. Chem..

[23] Greis K., Kirschbaum C., Leichnitz S., Gewinner S., Schöllkopf W., von Helden G., Meijer G., Seeberger P. H., Pagel K. (2020). Direct Experimental Characterization of the Ferrier Glycosyl Cation
in the Gas Phase. Org. Lett..

[24] Elferink H., Mensink R. A., Castelijns W. W. A., Jansen O., Bruekers J. P. J., Martens J., Oomens J., Rijs A. M., Boltje T. J. (2019). The Glycosylation
Mechanisms of 6,3-Uronic Acid Lactones. Angew.
Chem., Int. Ed..

[25] Hansen T., Elferink H., van Hengst J. M. A., Houthuijs K. J., Remmerswaal W. A., Kromm A., Berden G., van der
Vorm S., Rijs A. M., Overkleeft H. S., Filippov D. V., Rutjes F. P. J. T., van der Marel G. A., Martens J., Oomens J., Codée J. D. C., Boltje T. J. (2020). Characterization of Glycosyl Dioxolenium
Ions and Their Role in Glycosylation Reactions. Nat. Commun..

[26] Geue N., Greis K., Omoregbee-Leichnitz S., Kirschbaum C., Chang C.-W., Meijer G., von Helden G., Seeberger P. H., Pagel K. (2025). Influence of Levulinoyl Protecting
Groups on Glycosylation Stereoselectivity and Glycosyl Cation Structure. Eur. J. Org. Chem..

[27] Das R., Mukhopadhyay B. (2025). The Effect
of Neighbouring Group Participation and
Possible Long Range Remote Group Participation in O-Glycosylation. Beilstein J. Org. Chem..

[28] ter
Braak F., Houthuijs K. J., Elferink H., Kromm A., van Wieringen T., Berden G., Martens J., Oomens J., Boltje T. J. (2024). Investigation of Neighboring Group Participation in
3,4-Diacetylated Glycosyl Donors in the Gas Phase. Chem. Eur. J..

[29] Grabarics M., Lettow M., Kirschbaum C., Greis K., Manz C., Pagel K. (2022). Mass Spectrometry-Based
Techniques to Elucidate the Sugar Code. Chem.
Rev..

[30] Greis K., Griesbach C. E., Kirschbaum C., Meijer G., von Helden G., Pagel K., Peczuh M. W. (2023). Characterization
and Fate of a Septanosyl
Ferrier Cation in the Gas and Solution Phases. J. Org. Chem..

[31] Marianski M., Supady A., Ingram T., Schneider M., Baldauf C. (2016). Assessing the Accuracy of Across-the-Scale
Methods
for Predicting Carbohydrate Conformational Energies for the Examples
of Glucose and α-Maltose. J. Chem. Theory
Comput..

[32] de
Kleijne F. F. J., Elferink H., Moons S. J., White P. B., Boltje T. J. (2022). Characterization of Mannosyl Dioxanium Ions in Solution
Using Chemical Exchange Saturation Transfer NMR Spectroscopy. Angew. Chem., Int. Ed..

[33] Tomasi J., Mennucci B., Cammi R. (2005). Quantum Mechanical
Continuum Solvation
Models. Chem. Rev..

[34] Geue N., Greis K., Omoregbee-Leichnitz S., Kirschbaum C., Meijer G., von Helden G., Seeberger P. H., Marianski M., Pagel K. (2025). Evaluating C2-Neighbouring
and C3-,
C4-, C6-Remote Participation in Peracetylated Glycosyl Cations. ChemRxiv.

