# Supplementary material for: Evaluating Participation Modes in Peracetylated Glycosyl Cations
Source: Org Lett. 2026 Jan 2;28(14):4337–42. doi: 10.1021/acs.orglett.5c04756 (PMC13077679; doi:10.1021/acs.orglett.5c04756)
Supplement: Supplementary file 1 [file ol5c04756_si_001.pdf]

Supporting Information

Evaluating Participation Modes in Peracetylated Glycosyl Cations

Niklas Geue,[1,2,†] Kim Greis,[1,2,†] Sabrina Omoregbee-Leichnitz,[1,3] Carla Kirschbaum,[1,2] Gerard Meijer,[2] Gert von Helden,[2] Peter H. Seeberger,[1,3] Mateusz Marianski,[4] and Kevin Pagel[1,2,*]

[1]Institute of Chemistry and Biochemistry, Freie Universität Berlin, Altensteinstraße 23a, 14195 Berlin, Germany. [2]Department of Molecular Physics, Fritz-Haber-Institut der Max-Planck-Gesellschaft, Faradayweg 4–6, 14195 Berlin, Germany. [3]Max Planck Institute of Colloids and Interfaces, Am Mühlenberg 1, 14476 Potsdam, Germany. [4]Department of Chemistry, Hunter College, The City University of New York, New York, NY 10065, USA.

[†]These authors contributed equally.

[*]Corresponding author: kevin.pagel@fu-berlin.de

Table of Contents

Experimental Details 3

Mass Spectrometry and Gas-Phase Infrared Spectroscopy 3

Computational Methods 4

Supporting Figures and Tables 5

Figure S1: Mass spectra of a) **2,3,4,6-Ac-Glc**, b) **2,3,4,6-Ac-Gal** and c) **2,3,4,6-Ac-Man**. 5

Table S1: Energetics of **2,3,4,6-Ac-Glc** structures 6

Table S2: Energetics of **2,3,4,6-Ac-Gal** structures 8

Table S3: Energetics **2,3,4,6-Ac-Man** structures 10

Figure S2: Lowest energy structures for each structural motif of **2,3,4,6-Ac-Glc**. 12

Figure S3: Lowest energy structures for each structural motif of **2,3,4,6-Ac-Gal**. 13

Figure S4: Lowest energy structures for each structural motif of **2,3,4,6-Ac-Man**. 14

Figure S5: Impact of solvent on the stability of **2,3,4,6-Ac-Glc** ions *in silico*. 15

Figure S6: Impact of solvent on the stability of **2,3,4,6-Ac-Gal** ions *in silico*. 16

Figure S7: Impact of solvent on the stability of **2,3,4,6-Ac-Man** ions *in silico*. 17

References 18

Experimental Details

Mass Spectrometry and Gas-Phase Infrared Spectroscopy

Ethyl 2,3,4,6-tetra-*O*-acetyl-1-thio-α-ᴅ-glucopyranoside, ethyl 2,3,4,6-tetra-*O*-acetyl-1-thio-β-ᴅ-galactopyranoside and ethyl 2,3,4,6-tetra-*O*-acetyl-1-thio-α-ᴅ-mannopyranoside are commercially available from ChemPep, Synthose and Merck, respectively.

Samples were dissolved in a 9:1 (v/v) mixture of acetonitrile and water, with final concentrations of 0.1 mM. Pd/Pt coated glass capillaries (Sputter Coater HR 208, Cressington) were pulled to capillaries with an inner tip diameter of 1–2 µm using a micropipette puller (Model P-1000, Sutter Instrument) and used for nano electrospray ionization (nESI). Glycosyl cations were obtained *via* in-source fragmentation and measured using a custom-built helium droplet instrument, described elsewhere in detail.[1]

Samples are transferred to the gas phase with nESI using a capillary voltage of ca. 1 kV. Commonly, nESI of the glycan precursors leads to protonated, ammoniated and sodiated ions, although for labile leaving groups, in this case SEt, they can be cleaved by in-source fragmentation, resulting in the formation of the respective glycosyl cations. The glycosyl cations of interest are *m/z*-selected by a quadrupole mass filter before they enter a quadrupole bender. In the absence of additional voltage, the ions directly pass through the bender to be analysed in a time-of-flight (ToF) mass analyzer, resulting in the measurement of the mass (Figure S1). When a voltage is applied to the quadrupole bender, the ions are deflected and enter a hexapole ion trap that is cooled to 90 K by liquid nitrogen-cooled helium.

Pressurized helium expands into the vacuum through a pulsed Even-Lavie valve, which results in a beam of superfluid helium nanodroplets (0.4 K) passing through the ion trap. Glycosyl cations are picked up, cooled and guided to the infrared (IR) detection region, where an IR beam generated by the Fritz Haber Institute free-electron (FHI-FEL) laser interacts with the ion beam.[2] Resonant photons are absorbed, leading to vibrational excitation of the ions. The ions dissipate the energy to the helium matrix, relaxing to their vibrational ground state. The sequential absorption of multiple photons leads to the release of the ions from the helium nanodroplets, and detection in the ToF analyzer. The ion yield is plotted as a function of the IR wavenumber, leading to a gas-phase IR spectrum. Due to the nature of the multiphoton absorption process, the intensities in the experimental IR spectrum do not scale linearly across different energies/wavenumbers. As a first-order correction, the ion signal is divided by the energy of the IR macropulse.

Computational Methods

The genetic algorithm (GA) FAFOOM[3] was used to sample the conformational space of the **2,3,4,6-Ac-Glc**, **2,3,4,6-Ac-Gal** and **2,3,4,6-Ac-Man** building blocks, using the respective oxocarbenium ions as the starting points. After their generation, each structure was sent to the external software FHI-aims (version 171221)[4] for local DFT geometry optimization at the dispersion corrected PBE+vdW^{TS} level of theory[5,6] using *light* basis set settings for all atoms. For each glycosyl cation, multiple separate GA runs were carried out using the settings in the table below. The GA sampling of the glycosyl cations yielded structures with five different ion types: dioxolenium-type structures exhibiting neighbouring participation from C2, remote participation from the C3-, C4- or C6-acetyl protecting group as well as oxocarbenium-type structures without any participation. GA parameters used in initial search of glycosyl cations are shown below. Reproduced from the Supporting Information of Ref. 7.

	Parameter	Value
Molecule	Distance_cutoff_1	1.2
	Distance_cutoff_2	2.15
	Rmsd_cutoff_uniq	0.25
GA settings	Popsize	10
	Prob_for_crossing	0.95
	Prob_for_mut_pyranosering	0.6
	Prob_for_mut_torsion	0.8
	Fitness_sum_limit	1.2
	Selection	Roulette wheel
	Max_mutations_torsion	3
	Max_mutations_pyranosering	1

From all the structures generated by the GA, several low-energy structures for each ion type were selected for reoptimization and harmonic frequency calculation at the PBE0+D3/6-311+G(d,p) level of theory[8–10] in Gaussian 16, Revision A.03[11] using default settings. The energies including the zero-point vibrational energy (ZPVE) and free energies at 90 K (the estimated temperature in the hexapole ion trap) of the reoptimized structures are shown in Tables S1-S3. The computed IR spectra were normalized and scaled by an empirical factor of 0.965.[12]

For each building block, the energetically lowest gas-phase conformers of C2-, C3-, C4- and C6-dioxolenium ions, as well as oxocarbenium ions, were reoptimized (using the same settings as described in the previous paragraph) with an implicit solvation model (keyword: SCRF=Solvent)[13,14] in 1,4-dioxane (ε = 2.2099), toluene (ε = 2.3741), diethylether (ε = 4.2400), chloroform (ε = 4.7113), dichlormethane (ε = 8.93), acetonitrile (ε = 35.688), dimethylsulfoxide (ε = 46.826) and water (ε = 78.3553). Relative electronic energies were plotted as a function of the respective dielectric constants (Figures S5 – S7).

Supporting Figures and Tables

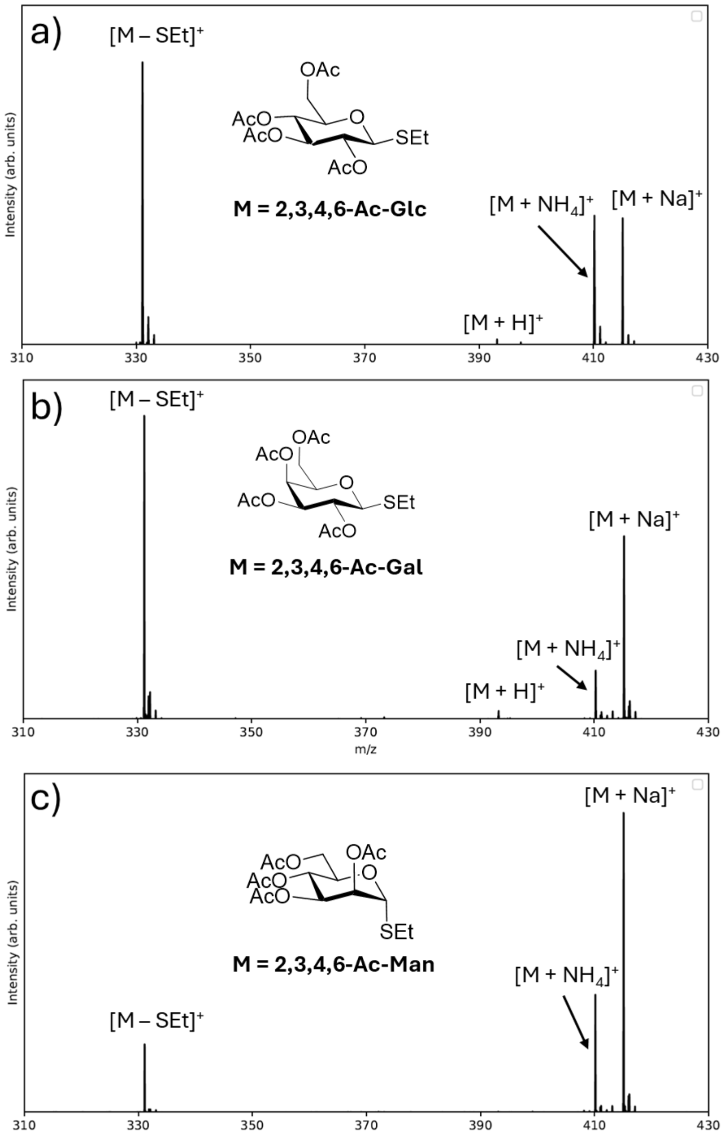

Figure S1: Mass spectra of a) **2,3,4,6-Ac-Glc**, b) **2,3,4,6-Ac-Gal** and c) **2,3,4,6-Ac-Man**. β-thioethyl (SEt) was used as a leaving group for each precursor (M). The respective glycosyl cations [M – SEt]+ are obtained *via* in-source fragmentation.

Table S1: Energetics of **2,3,4,6-Ac-Glc** structures optimized at the PBE0+D3/6-311+G(d,p) level of theory. Energies (ΔE, including zero-point-vibrational energy) and free energies (ΔF) at 90 K are assigned to each structure. The infrared spectra of the structures labelled with an asterisk are represented in the manuscript.

ID	ΔE(PBE0 + D3) [kJ mol^{-1}]	ΔF(PBE0 + D3) [kJ mol^{-1}]
C2_dioxolenium/conf_00*	0.00	0.00
C2_dioxolenium/conf_01	7.92	7.07
C2_dioxolenium/conf_02	2.22	1.63
C2_dioxolenium/conf_03	3.36	1.99
C2_dioxolenium/conf_04	2.22	1.65
C2_dioxolenium/conf_05	10.69	9.00
C2_dioxolenium/conf_06	3.36	3.73
C2_dioxolenium/conf_07	3.90	3.97
C2_dioxolenium/conf_08	15.71	13.49
C2_dioxolenium/conf_09	8.64	7.70
C2_dioxolenium/conf_10	10.69	9.01
C2_dioxolenium/conf_11	12.02	9.70
C2_dioxolenium/conf_12	8.93	8.21
C2_dioxolenium/conf_13	11.25	9.59
C2_dioxolenium/conf_14	8.92	8.18
C3_dioxolenium/conf_00	33.53	30.56
C3_dioxolenium/conf_01*	27.42	25.65
C3_dioxolenium/conf_02	29.72	28.71
C3_dioxolenium/conf_03	29.58	28.59
C3_dioxolenium/conf_04	33.14	32.03
C3_dioxolenium/conf_05	33.92	31.70
C3_dioxolenium/conf_06	40.12	37.89
C4_dioxolenium/conf_00	31.41	31.34
C4_dioxolenium/conf_01*	29.84	28.79
C4_dioxolenium/conf_02	31.17	29.64
C4_dioxolenium/conf_03	34.79	32.36
C4_dioxolenium/conf_04	35.56	32.74
C4_dioxolenium/conf_05	38.61	36.03

C4_dioxolenium/conf_06	31.41	31.32
C4_dioxolenium/conf_07	36.52	32.95
C6_dioxolenium/conf_00*	41.81	39.31
C6_dioxolenium/conf_01	43.97	42.80
C6_dioxolenium/conf_02	44.25	42.19
C6_dioxolenium/conf_03	44.91	43.04
C6_dioxolenium/conf_04	48.53	46.42
C6_dioxolenium/conf_05	48.66	46.34
C6_dioxolenium/conf_06	53.46	49.44
C6_dioxolenium/conf_07	51.56	51.39
C6_dioxolenium/conf_08	55.78	54.80
C6_dioxolenium/conf_09	53.47	49.47
oxocarbenium/conf_00*	59.95	55.74
oxocarbenium/conf_01	60.44	55.78
oxocarbenium/conf_02	60.44	55.79
oxocarbenium/conf_03	67.93	64.29
oxocarbenium/conf_04	70.46	66.60
oxocarbenium/conf_05	61.79	58.57
oxocarbenium/conf_06	73.93	69.20
oxocarbenium/conf_07	80.56	77.16

Table S2: Energetics of **2,3,4,6-Ac-Gal** structures optimized at the PBE0+D3/6-311+G(d,p) level of theory. Energies (ΔE, including zero-point-vibrational energy) and free energies (ΔF) at 90 K are assigned to each structure. The infrared spectra of the structures labelled with an asterisk are represented in the manuscript.

ID	ΔE(PBE0 + D3) [kJ mol^{-1}]	ΔF(PBE0 + D3) [kJ mol^{-1}]
C2_dioxolenium/conf_00	-0.15	1.25
C2_dioxolenium/conf_01	0.00	0.00
C2_dioxolenium/conf_02	1.41	2.00
C2_dioxolenium/conf_03	4.00	3.22
C2_dioxolenium/conf_04	2.27	0.98
C2_dioxolenium/conf_05	2.32	0.73
C2_dioxolenium/conf_06	5.47	4.79
C2_dioxolenium/conf_07	7.55	6.70
C2_dioxolenium/conf_08	5.47	4.75
C2_dioxolenium/conf_09*	6.31	7.84
C2_dioxolenium/conf_M00	-0.16	1.24
C2_dioxolenium/conf_M01	0.01	0.06
C2_dioxolenium/conf_M02	4.01	3.24
C2_dioxolenium/conf_M03*	1.88	0.49
C2_dioxolenium/conf_M04	11.44	13.72
C3_dioxolenium/conf_00*	16.69	17.39
C3_dioxolenium/conf_01	21.80	24.46
C3_dioxolenium/conf_02	25.91	28.03
C3_dioxolenium/conf_03	34.34	32.55
C3_dioxolenium/conf_04	21.80	24.48
C3_dioxolenium/conf_M00	25.90	28.02
C3_dioxolenium/conf_M01	40.61	40.74
C3_dioxolenium/conf_M02	51.89	55.22
C3_dioxolenium/conf_M03	70.12	72.03
C4_dioxolenium/conf_00*	9.27	10.65
C4_dioxolenium/conf_01	18.87	20.47
C4_dioxolenium/conf_02	15.92	16.38
C4_dioxolenium/conf_03	14.91	16.71

C4_dioxolenium/conf_04	14.39	13.65
C4_dioxolenium/conf_05	17.92	18.17
C4_dioxolenium/conf_06	16.21	17.68
C4_dioxolenium/conf_07	16.44	17.63
C4_dioxolenium/conf_M00	15.92	16.38
C4_dioxolenium/conf_M01	16.73	16.37
C4_dioxolenium/conf_M02	23.18	22.77
C6_dioxolenium/conf_00*	25.66	27.39
C6_dioxolenium/conf_01	34.38	37.22
C6_dioxolenium/conf_02	35.21	35.63
C6_dioxolenium/conf_03	34.68	35.49
C6_dioxolenium/conf_04	51.58	51.85
C6_dioxolenium/conf_05	70.26	71.98
C6_dioxolenium/conf_M00	25.66	27.39
C6_dioxolenium/conf_M01	50.30	52.25
C6_dioxolenium/conf_M02	53.02	54.83
oxocarbenium/conf_00*	60.52	59.04
oxocarbenium/conf_01	61.53	59.58
oxocarbenium/conf_02	61.62	59.37
oxocarbenium/conf_03	64.24	63.98
oxocarbenium/conf_04	64.98	62.62
oxocarbenium/conf_M00	61.53	59.57
oxocarbenium/conf_M01	87.38	85.96
oxocarbenium/conf_M02	88.64	87.67
oxocarbenium/conf_M03	76.40	77.26

Table S3: Energetics **2,3,4,6-Ac-Man** structures optimized at the PBE0+D3/6-311+G(d,p) level of theory. Energies (ΔE, including zero-point-vibrational energy) and free energies (ΔF) at 90 K are assigned to each structure. The infrared spectra of the structures labelled with an asterisk are represented in the manuscript.

**ID**	**ΔE(PBE0 + D3) [kJ mol^{-1}]**	**ΔF(PBE0 + D3) [kJ mol^{-1}]**
C2_dioxolenium/conf_00*	0.00	0.00
C2_dioxolenium/conf_01	0.00	0.01
C2_dioxolenium/conf_02	5.87	6.45
C2_dioxolenium/conf_03*	5.87	6.44
C2_dioxolenium/conf_04*	3.79	2.01
C2_dioxolenium/conf_05	9.43	7.54
C2_dioxolenium/conf_06	15.77	14.32
C2_dioxolenium/conf_07	15.77	14.31
C2_dioxolenium/conf_08	19.94	17.73
C2_dioxolenium/conf_09	19.79	18.95
C2_dioxolenium/conf_10	15.60	16.01
C2_dioxolenium/conf_11	17.24	18.37
C2_dioxolenium/conf_12	10.68	9.77
C2_dioxolenium/conf_13	10.68	9.78
C2_dioxolenium/conf_14	12.53	11.72
C3_dioxolenium/conf_00*	9.42	7.48
C3_dioxolenium/conf_01	12.15	10.35
C3_dioxolenium/conf_02	8.89	7.65
C3_dioxolenium/conf_03	9.54	7.79
C3_dioxolenium/conf_04	9.54	7.78
C3_dioxolenium/conf_05	18.81	17.64
C3_dioxolenium/conf_06	15.03	13.40
C3_dioxolenium/conf_07	16.44	15.89
C3_dioxolenium/conf_08	15.97	15.53
C4_dioxolenium/conf_00*	37.64	36.97
C4_dioxolenium/conf_01	40.31	39.44
C4_dioxolenium/conf_02	42.86	40.94
C4_dioxolenium/conf_03	45.86	44.22

C4_dioxolenium/conf_04	71.82	72.13
C4_dioxolenium/conf_05	76.95	76.59
C4_dioxolenium/conf_06	81.04	82.53
C6_dioxolenium/conf_00	36.05	35.88
C6_dioxolenium/conf_01	42.37	44.27
C6_dioxolenium/conf_02	45.94	46.17
C6_dioxolenium/conf_03*	36.05	35.87
C6_dioxolenium/conf_04	64.30	64.88
C6_dioxolenium/conf_05	65.90	66.97
oxocarbenium/conf_00*	64.69	60.75
oxocarbenium/conf_01	63.58	59.52
oxocarbenium/conf_02	68.82	65.23
oxocarbenium/conf_03	70.04	65.34
oxocarbenium/conf_04	69.51	67.40
oxocarbenium/conf_05	70.62	69.03
oxocarbenium/conf_06	68.86	65.00

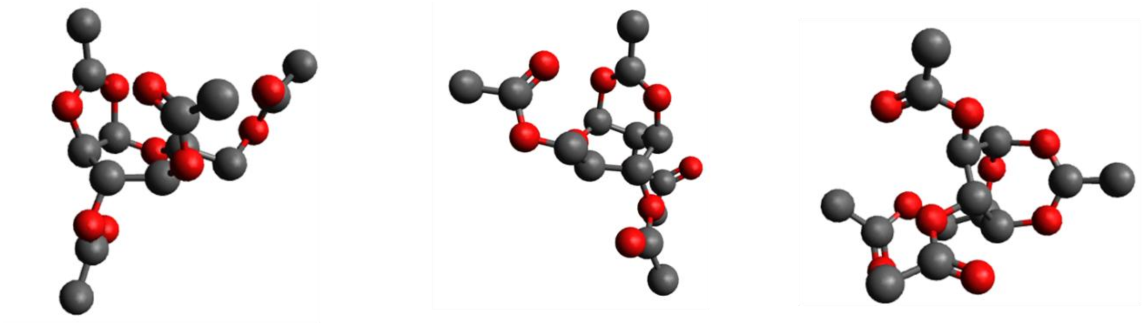

C2_dioxolenium **C3_dioxolenium** **C4_dioxolenium**

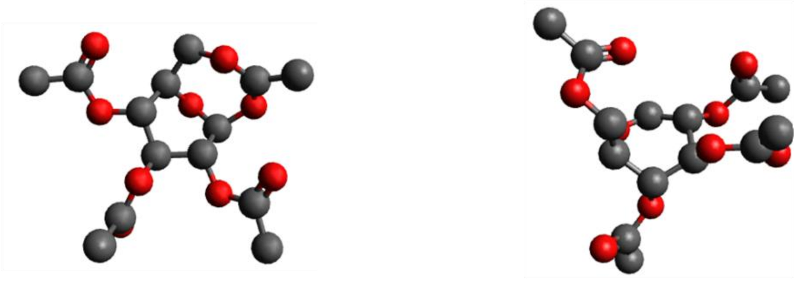

C6_dioxolenium **oxocarbenium**

Figure S2: Lowest energy structures for each structural motif of **2,3,4,6-Ac-Glc**. Hydrogen atoms are omitted for clarity.

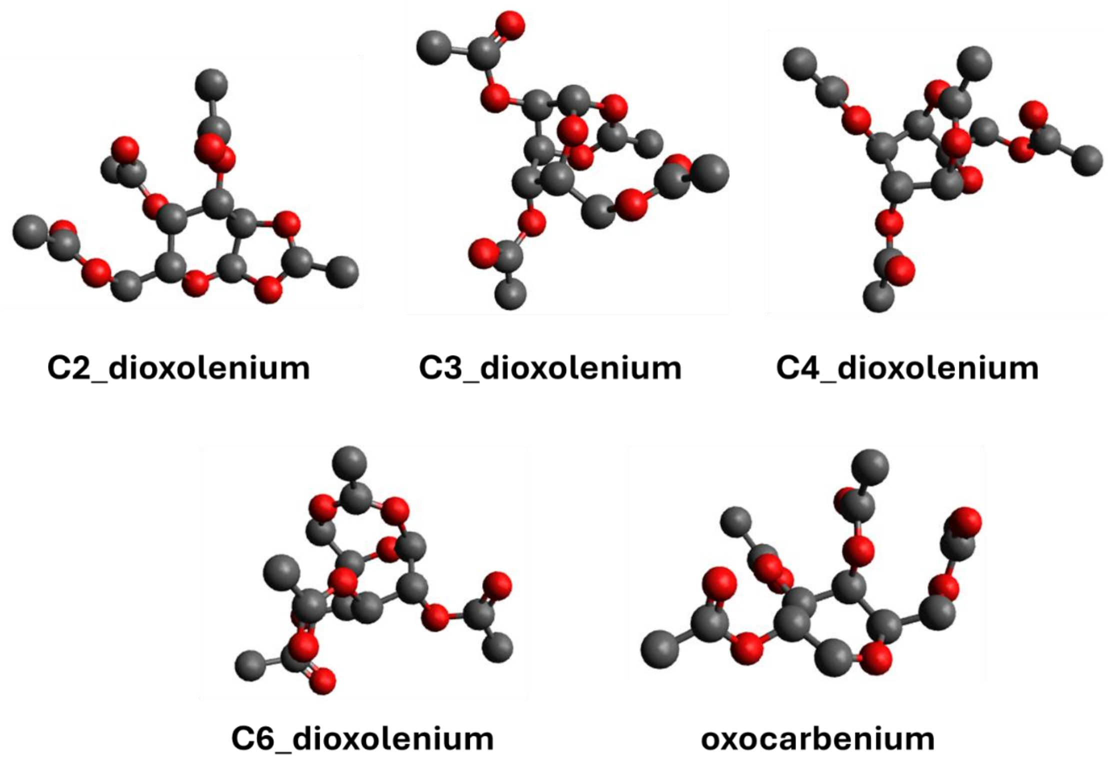

Figure S3: Lowest energy structures for each structural motif of **2,3,4,6-Ac-Gal**. Hydrogen atoms are omitted for clarity.

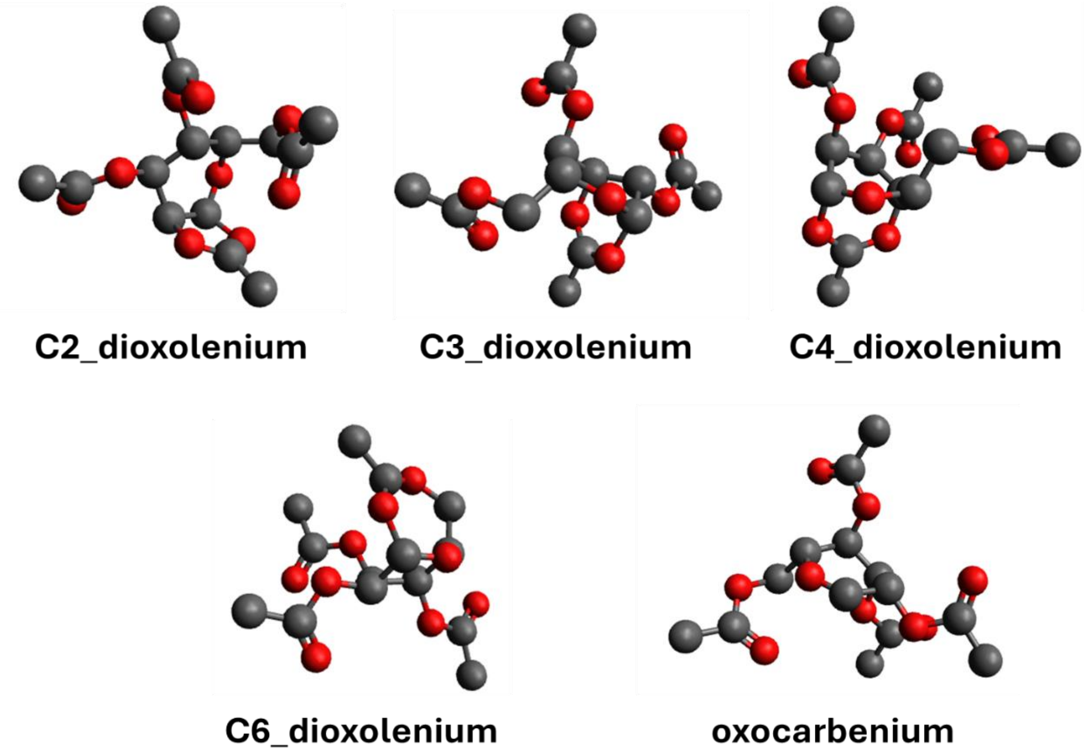

Figure S4: Lowest energy structures for each structural motif of **2,3,4,6-Ac-Man**. Hydrogen atoms are omitted for clarity.

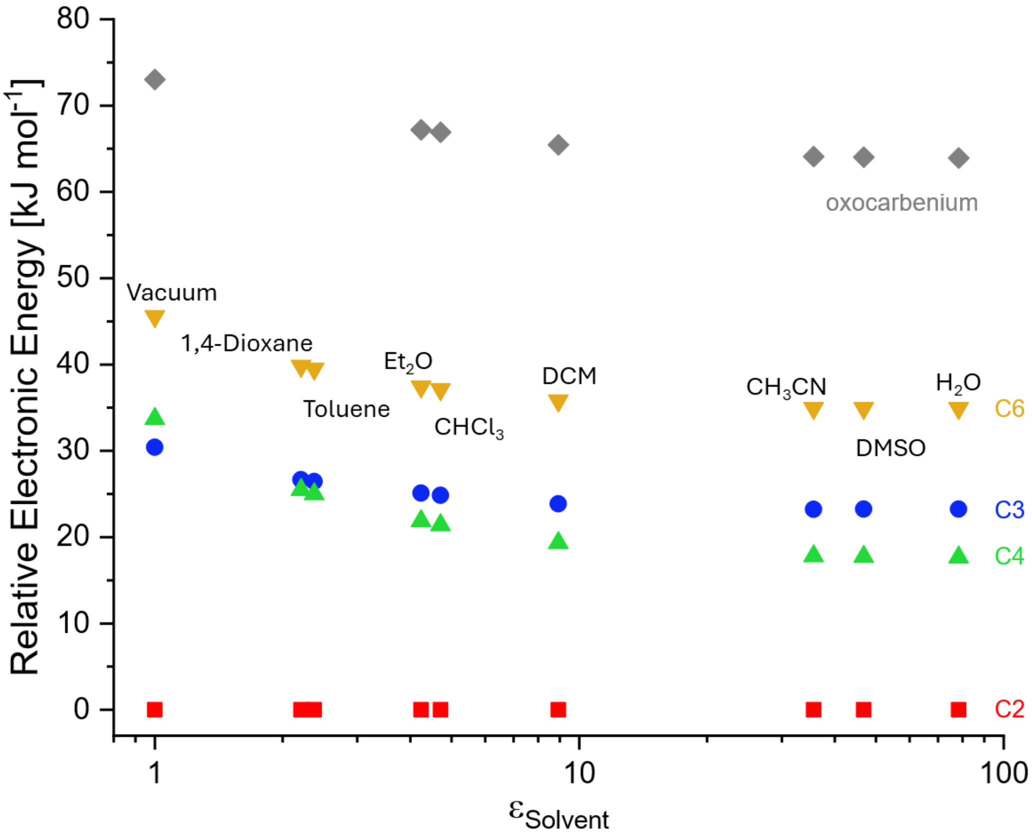

Figure S5: Impact of solvent on the stability of **2,3,4,6-Ac-Glc** ions *in silico*. Relative electronic energies of the C2- (red), C3- (blue), C4- (green), and C6-dioxolenium ions (yellow) as well as oxocarbenium ions (grey) for **2,3,4,6-Ac-Glc**, optimized with implicit solvation models, are plotted as a function of the dielectric constant. The oxocarbenium ions could not be optimized in 1,4-dioxane and toluene. The relative electronic energies can be found in the Supplementary Dataset.

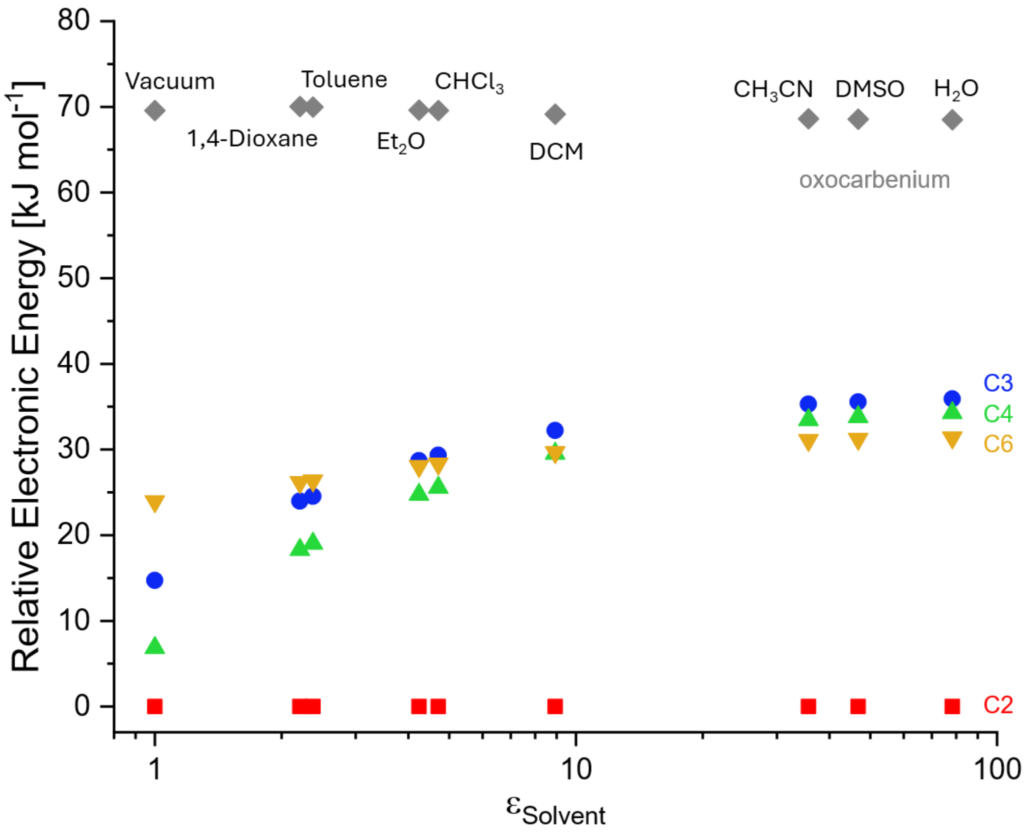

Figure S6: Impact of solvent on the stability of **2,3,4,6-Ac-Gal** ions *in silico*. Relative electronic energies of the C2- (red), C3- (blue), C4- (green), and C6-dioxolenium ions (yellow) as well as oxocarbenium ions (grey) for **2,3,4,6-Ac-Gal**, optimized with implicit solvation models, are plotted as a function of the dielectric constant. The relative electronic energies can be found in the Supplementary Dataset.

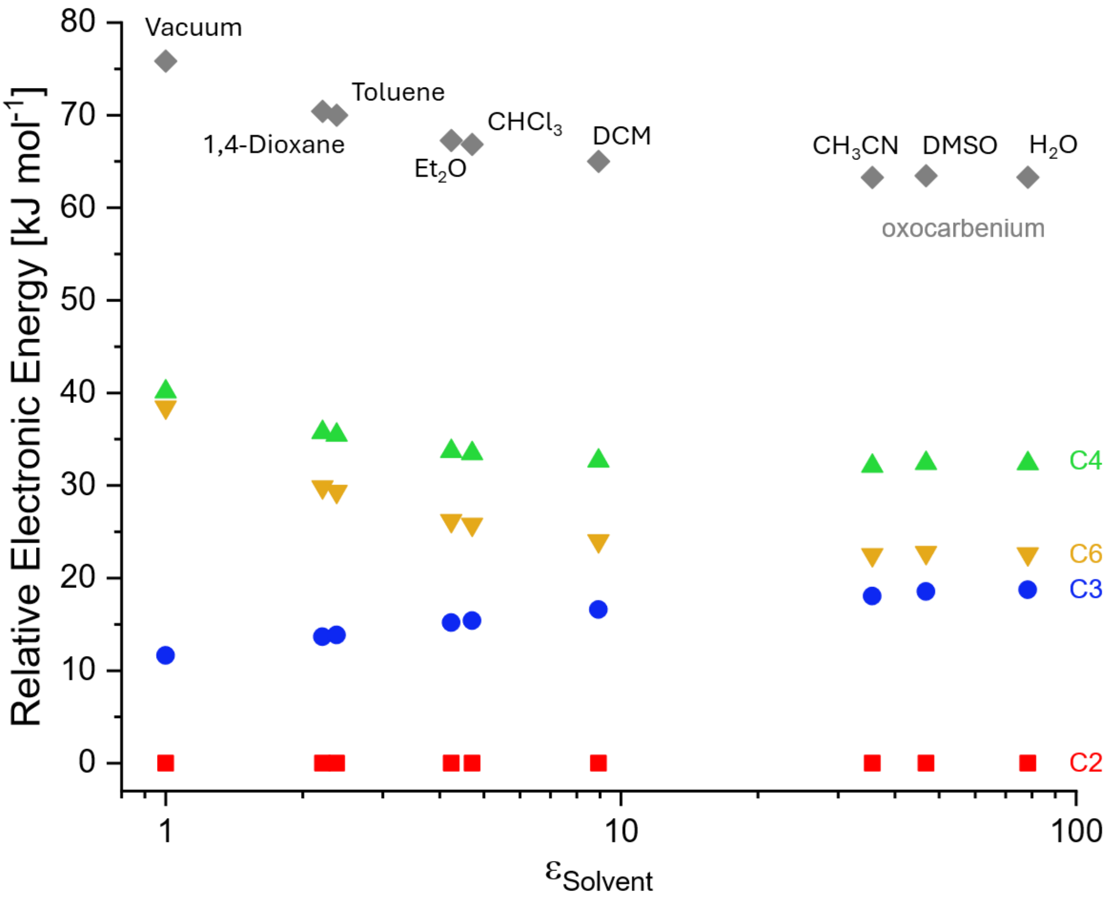

Figure S7: Impact of solvent on the stability of **2,3,4,6-Ac-Man** ions *in silico*. Relative electronic energies of the C2- (red), C3- (blue), C4- (green), and C6-dioxolenium ions (yellow) as well as oxocarbenium ions (grey) for **2,3,4,6-Ac-Man**, optimized with implicit solvation models, are plotted as a function of the dielectric constant. The relative electronic energies can be found in the Supplementary Dataset.

References

(1) Thomas, D. A.; Chang, R.; Mucha, E.; Lettow, M.; Greis, K.; Gewinner, S.; Schöllkopf, W.; Meijer, G.; Helden, G. von. Probing the Conformational Landscape and Thermochemistry of DNA Dinucleotide Anions via Helium Nanodroplet Infrared Action Spectroscopy. *Phys. Chem. Chem. Phys.* **2020**, *22* (33), 18400–18413. https://doi.org/10.1039/D0CP02482A.

(2) Schöllkopf, W.; Gewinner, S.; Junkes, H.; Paarmann, A.; Helden, G. von; Bluem, H. P.; Todd, A. M. M. The New IR and THz FEL Facility at the Fritz Haber Institute in Berlin. In *Advances in X-ray Free-Electron Lasers Instrumentation III*; SPIE, 2015; Vol. 9512, pp 238–250. https://doi.org/10.1117/12.2182284.

(3) Supady, A.; Blum, V.; Baldauf, C. First-Principles Molecular Structure Search with a Genetic Algorithm. *J. Chem. Inf. Model.* **2015**, *55* (11), 2338–2348. https://doi.org/10.1021/acs.jcim.5b00243.

(4) Blum, V.; Gehrke, R.; Hanke, F.; Havu, P.; Havu, V.; Ren, X.; Reuter, K.; Scheffler, M. *Ab Initio* Molecular Simulations with Numeric Atom-Centered Orbitals. *Comput. Phys. Commun.* **2009**, *180* (11), 2175–2196. https://doi.org/10.1016/j.cpc.2009.06.022.

(5) Perdew, J. P.; Burke, K.; Ernzerhof, M. Generalized Gradient Approximation Made Simple. *Phys. Rev. Lett.* **1996**, *77* (18), 3865–3868. https://doi.org/10.1103/PhysRevLett.77.3865.

(6) Tkatchenko, A.; Scheffler, M. Accurate Molecular Van Der Waals Interactions from Ground-State Electron Density and Free-Atom Reference Data. *Phys. Rev. Lett.* **2009**, *102* (7), 073005. https://doi.org/10.1103/PhysRevLett.102.073005.

(7) Greis, K.; Leichnitz, S.; Kirschbaum, C.; Chang, C.-W.; Lin, M.-H.; Meijer, G.; von Helden, G.; Seeberger, P. H.; Pagel, K. The Influence of the Electron Density in Acyl Protecting Groups on the Selectivity of Galactose Formation. *J. Am. Chem. Soc.* **2022**, *144* (44), 20258–20266. https://doi.org/10.1021/jacs.2c05859.

(8) Adamo, C.; Barone, V. Toward Reliable Density Functional Methods without Adjustable Parameters: The PBE0 Model. *J. Chem. Phys.* **1999**, *110* (13), 6158–6170. https://doi.org/10.1063/1.478522.

(9) Grimme, S.; Antony, J.; Ehrlich, S.; Krieg, H. A Consistent and Accurate Ab Initio Parametrization of Density Functional Dispersion Correction (DFT-D) for the 94 Elements H-Pu. *J. Chem. Phys.* **2010**, *132* (15), 154104. https://doi.org/10.1063/1.3382344.

(10) Hehre, W. J.; Ditchfield, R.; Pople, J. A. Self—Consistent Molecular Orbital Methods. XII. Further Extensions of Gaussian—Type Basis Sets for Use in Molecular Orbital Studies of Organic Molecules. *J. Chem. Phys.* **1972**, *56* (5), 2257–2261. https://doi.org/10.1063/1.1677527.

(11) Frisch, M. J.; Trucks, G. W.; Schlegel, H. B.; Scuseria, G. E. ; Robb, M. A. ; Cheeseman, J. R. ; Scalmani, G. ; Barone, V. ; Petersson, G. A. ; Nakatsuji, H. ; Li, X. ; Caricato, M. ; Marenich, A. V. ; Bloino, J. ; Janesko, B. G. ; Gomperts, R. ; Mennucci, B. ; Hratchian, H. P. ; Ortiz, J. V. ; Izmaylov, A. F. ; Sonnenberg, J. L. ; Williams-Young, D. ; Ding, F. ; Lipparini, F. ; Egidi, F. ; Goings, J. ; Peng, B. ; Petrone, A. ; Henderson, T. ; Ranasinghe, D. ; Zakrzewski, V. G. ; Gao, J. ; Rega, N. ; Zheng, G. ; Liang, W. ; Hada, M. ; Ehara, M. ; Toyota, K. ; Fukuda, R. ; Hasegawa, J. ; Ishida, M. ; Nakajima, T. ; Honda, Y. ; Kitao, O. ; Nakai, H. ; Vreven, T. ; Throssell, K. ; Montgomery, J. A. Jr. ; Peralta, J. E. ; Ogliaro, F. ; Bearpark, M. J. ; Heyd, J. J. ; Brothers, E. N. ; Kudin, K. N. ; Staroverov, V. N. ; Keith, T. A. ; Kobayashi, R. ; Normand, J. ; Raghavachari, K. ; Rendell, A. P. ; Burant, J. C. ; Iyengar, S. S. ; Tomasi, J. ; Cossi, M. ; Millam, J. M. ; Klene, M. ; Adamo, C. ; Cammi, R. ; Ochterski, J. W. ; Martin, R. L. ; Morokuma, K. ; Farkas, O. ; Foresman, J. B. ;; Fox, D. J. Gaussian 16, Revision C.01, 2016.

(12) Marianski, M.; Mucha, E.; Greis, K.; Moon, S.; Pardo, A.; Kirschbaum, C.; Thomas, D. A.; Meijer, G.; von Helden, G.; Gilmore, K.; Seeberger, P. H.; Pagel, K. Remote Participation during Glycosylation Reactions of Galactose Building Blocks: Direct Evidence from

Cryogenic Vibrational Spectroscopy. *Angew. Chem. Int. Ed.* **2020**, *59* (15), 6166–6171. https://doi.org/10.1002/anie.201916245.
(13) Tomasi, J.; Mennucci, B.; Cammi, R. Quantum Mechanical Continuum Solvation Models. *Chem. Rev.* **2005**, *105* (8), 2999–3094. https://doi.org/10.1021/cr9904009.
(14) Scalmani, G.; Frisch, M. J. Continuous Surface Charge Polarizable Continuum Models of Solvation. I. General Formalism. *J. Chem. Phys.* **2010**, *132* (11), 114110. https://doi.org/10.1063/1.3359469.
